# Evaluation of Regional Ecological Efficiency and Intelligent Decision Support for Sustainable Development Based on Environmental Big Data

**DOI:** 10.1155/2022/2820426

**Published:** 2022-01-27

**Authors:** Lelai Shi, Jing Zhou, Xingchi Zhou

**Affiliations:** ^1^Center for Industrial Economic Studies, School of Economics, Wuhan Textile University, Wuhan 430200, China; ^2^School of Management, Wuhan Textile University, Wuhan 430200, China

## Abstract

To promote urbanization in the next stage, it is of great significance to explore the ecological efficiency of green ecological regions and advance the sustainable development of a social economic system. However, spatial heterogeneity has not been fully considered in the existing evaluation models or methods for regional ecological efficiency (REE), and the corresponding decisions on sustainable development are not the optimal solutions. To solve the problems, this paper explores the evaluation of REE and intelligent decision support for sustainable development by analyzing environmental big data. Firstly, the spatiotemporal evolution of REE was examined based on environmental big data to clarify the spatial layout of REE and the sources of the spatial differences. Next, a multiobjective optimal decision-making model was established for the sustainable development of a regional ecosystem, and the solving method was presented for the model. The proposed model was proved valid through experiments.

## 1. Introduction

Urban development faces several problems, namely, the development model is extensive, the newly developed areas are underpopulated, and the resources are in short supply. With the development of the industry, these problems have intensified the contradiction between population, environment, and resources [1–5]. Rather than pursuing the single goal of economic interest, the sustainable development of the urbanization system aims to organize production for the composite goal of resources/energy, environment, economy, and society.

China is vigorously implementing a strategy of regional ecosystem development and environmental protection, which will continuously enhance the coordination between the regional environment, regional economy, and regional society [6–12]. Against this backdrop, in order to promote urbanization in the next stage, it is of great significance to explore the ecological efficiency of green ecological regions and advance the sustainable development of the social economic system [13–21].

Environmental pollution is a potential bottleneck of stable economic growth. As an effective measure of sustainable development, ecological efficiency can fully reflect the actual level of coordinated development between economy and environment. Liu and Sun [22] constructed a data envelopment analysis (DEA) model with environmental pollution and resource consumption as inputs and total economy as the output. Ratner [23] described the monitoring of ecological and economic efficiencies of the activities in the regional economic system, compared the applicability of several methods to the dynamic DEA model, and attempted to generate time series based on environmental and economic efficiency points. To evaluate the performance of the urban system, Giordano et al. [24] divided the urban system into traffic subsystem, built environment subsystem, and social economy subsystem, evaluated the efficiency of each subsystem by Takagi–Sugeno model, and recursively derived the overall efficiency of the entire system. Hoang and Alauddin [25] designed an input-oriented DEA framework, which allows the measurement and decomposition of economic, environmental, and ecological efficiencies in agricultural production of different countries.

On the sustainable development of the urban ecological system, Wang and Li [26] set up an overall framework for the sustainable urban spatial development model based on smart cities. The framework, involving such four dimensions as the monitoring, collection, interconnection, and sharing of urban data, provides an important guide for the spatial planning and construction of smart cities. Ma et al. [27] analyzed the evolution of an urban ecosystem and its dissipation structure and constructed an evaluation index system (EIS) for the sustainable development capability of the urban ecosystem.

The existing studies provide some useful references. However, the evaluation models and methods for regional ecological efficiency fail to fully consider spatial heterogeneity, take account of the dynamic evolution of ecological efficiency distribution, and thoroughly discuss the key factors affecting the ecological efficiency identification and animal habitat changes in different regions and at different levels. Concerning sustainable development, the available decision-making models overlook the balance of relative development for the regional ecosystem in spatiotemporal evolution and cannot converge to the optimal decisions. To solve the problems, this paper explores the evaluation of REE and intelligent decision support for sustainable development based on environmental big data.  Section 2 analyzes the spatiotemporal evolution of REE based on environmental big data, identifies the spatial layout and spatial difference of REEs, and discusses the key environmental factors.  Section 3 establishes a multiobjective optimal decision-making model for the sustainable development of the regional ecosystem and presents the solving method for the model. Experimental results demonstrate the effectiveness of our model.

This research discusses the inherent linking mechanism of system sustainable development with environmental, economic, and social impacts, and optimizes the absolute performance of sustainability and relative development simultaneously.

## 2. Spatiotemporal Evolution Analysis

This paper identifies the sources of environmental data for the spatiotemporal evolution of REE.  Table 1 lists the REE evaluation items based on big data. Several environmental types are enumerated, namely, building area, traffic area, water area, and landscape area, and the corresponding factors that affect ecological efficiency are presented in detail.

As mentioned before, this paper aims to clarify the spatial layout of REE and the sources of the spatial differences and realize the REE evaluation and make intelligent decisions for sustainable development. For this purpose, the spatial distribution of ecological efficiency was described intuitively based on environmental big data. Then, the degree of spatial differences of REE was investigated, and the sources of these differences were revealed. Finally, the dynamic evolution law of REE was explored through kernel density estimation (KDE) and Markov chain.

### 2.1. Spatial Difference Analysis

Through Dagum's decomposition of the Gini coefficient, this paper analyzes the spatial differences of global and local ecological efficiencies of the ecosystem in the study area. Dagum's decomposition method was selected for its capability of considering the overlap between subsamples, solving heterogeneous sources of REE spatial differences, and measuring how much overall regional differences are affected by the intraregional difference, inter-regional difference, and transvariation intensity.

Let *b*_*uv*_ and *b*_*gs*_ be the urban ecological efficiencies of the *u*-th and *g*-th regions, respectively; let *λ* be the mean ecological efficiency of all cities in a region, *m* be the total number of samples (cities), *l* be the number of regions, and *m*_*u*_ and *m*_*g*_ be the number of cities in the *u*-th and *g*-th regions, respectively. Then, the relative difference between all cities in a region in ecological efficiency can be measured by the regional overall Gini coefficient:

(1) $$\begin{array}{c} \text{GN}=\frac{\sum_{u=1}^{l}\sum_{g=1}^{l}\sum_{v=1}^{m_{u}}\sum_{s=1}^{m_{g}}\left|b_{uv}-b_{gs}\right|}{2m^{2}\lambda }. \end{array}$$

To decompose the Gini coefficient by a regional subsystem, the first step is to sort all cities in a region by mean ecological efficiency is as follows:

(2) $$\begin{array}{c} \lambda_{1}\leq \lambda_{2}\leq \cdots \leq \lambda_{g}\leq \cdots \leq \lambda_{u}\leq \cdots \leq \lambda_{l}. \end{array}$$

Ecological efficiency is the ratio of ecological outputs to ecological inputs. The outputs refer to the value of products and services provided by enterprises or economies, while the inputs refer to the resources and energies consumed by enterprises or economies, as well as the environmental load caused by enterprises or economies. The Gini coefficient of the *u*-th region can be calculated by

(3) $$\begin{array}{c} {\text{GN}}_{u}=\frac{\left(1/2\lambda_{u}\right)\sum_{v=1}^{m_{u}}\sum_{s=1}^{m_{u}}\left|b_{uv}-b_{us}\right|}{m_{u}^{2}}. \end{array}$$

The intraregional contribution difference *H*_*q*_ can be calculated by

(4) $$\begin{array}{c} H_{q}=\sum_{u=1}^{l}{\text{GN}}_{u}t_{u}. \end{array}$$

The inter-regional Gini coefficient GN_*ug*_ between the *u*-th and *g*-th regions can be calculated by

(5) $$\begin{array}{c} {\text{GN}}_{ug}=\frac{\sum_{v=1}^{m_{u}}\sum_{s=1}^{m_{g}}\left|b_{uv}-b_{gs}\right|}{m_{u}m_{g}\left(\lambda_{u}+\lambda_{g}\right)}. \end{array}$$

The inter-regional contribution difference *H*_*mr*_ can be calculated by

(6) $$\begin{array}{c} H_{mr}=\sum_{u=2}^{l}\sum_{h=2}^{u-1}{\text{GN}}_{ug}\left(t_{u}e_{h}+t_{h}e_{u}\right)C_{ug}. \end{array}$$

The transvariation intensity contribution *H*_*d*_ can be calculated by

(7) $$\begin{array}{c} H_{d}=\sum_{u=2}^{l}\sum_{g=2}^{u-1}{\text{GN}}_{ug}\left(t_{u}e_{g}+t_{g}e_{u}\right)\left(1-C_{ug}\right), \end{array}$$

where *e*_*i*_ = *m*_*i*_*λ*_*i*_/(*mλ*) and *t*_*j*_ = *m*_*j*_/*m*. Let *c*_*ug*_ be the ecological efficiency difference between regions; *t*_*ug*_ be the first moment of transvariation. Then, the relative influence *C*_*ug*_ of the unit ecological efficiency of the *u*-th and *g*-th regions can be calculated by

(8) $$\begin{array}{c} C_{ug}=\frac{c_{ug}-t_{ug}}{c_{ug}+t_{ug}}. \end{array}$$

Let *O*_*u*_ and *O*_*g*_ be the cumulative density distribution functions of the *u*-th and *g*-th regions, respectively. Then, *c*_*ug*_ can be characterized by the mathematical expectation of the sum of the positive sample values for the difference between the ecological efficiencies, *b*_*uv*_ and *b*_*gs*_, of the cities in the *u*-th and *g*-th regions:

(9) $$\begin{array}{c} c_{ug}=\int_{0}^{\infty }\text{d}O_{u}\left(b\right)\int_{0}^{y}\left(b-a\right)\text{d}O_{g}\left(a\right). \end{array}$$

In addition, *t*_*ug*_ can be calculated by

(10) $$\begin{array}{c} t_{ug}=\int_{0}^{\infty }\text{d}O_{g}\left(b\right)\int_{0}^{y}\left(b-a\right)\text{d}O_{u}\left(b\right). \end{array}$$

### 2.2. Dynamic Evolution of Distribution

The traditional KDE, a popular tool in spatial nonequilibrium analysis, is a nonparametric estimation approach. It can describe the form of each random variable with a continuous curve. In this way, virtually no statistical error will be incurred by the improper setting of ecological efficiencies in the region. Let *M* be the total number of cities in the region, *A*_*i*_ be the independent identically distributed observations, *a*^*∗*^ be the mean value, *τ* be the bandwidth, and Γ(·) be the kernel function. Then, the density function *μ*(*a*) of the random variable *A* can be calculated by

(11) $$\begin{array}{c} \mu \left(a\right)=\frac{1}{M\tau }\sum_{i=1}^{M}\Gamma \left\{\frac{\left(A_{i}-a^{*}\right)}{\tau }\right\}. \end{array}$$

The greater the *τ* value, the larger the neighbourhood of a. If *τ* is too large, the kernel density function Γ(·) will be too smooth. Then, some important features of the function will be smoothened out, causing a large deviation. Hence, the *τ* value should be minimized if possible. Thus, Γ(·) needs to meet the following condition:

(12) $$\begin{array}{c} \left\{\begin{array}{c} \underset{a⟶\infty }{\lim}\Gamma \left(a\right)\cdot a=0,\\ \Gamma \left(a\right)\geq 0,\;\int_{-\infty }^{+\infty }\Gamma \left(a\right)\text{d}a=1,\\ \sup\;\Gamma \left(x\right)<+\infty ,\;\int_{-\infty }^{+\infty }\Gamma \left(a\right)\text{d}a<+\infty . \end{array}\right) \end{array}$$

The dynamic evolution features of REE distribution can be estimated by the Gaussian kernel function:

(13) $$\begin{array}{c} \Gamma \left(a\right)=\frac{1}{\sqrt{2\pi }}e^{-a^{2}/2}. \end{array}$$

### 2.3. Long-Term Transition Trend

This paper adopts the Markov chain to describe the ecological efficiency of each city in a region, that is, sets up the transition probability matrix of the Markov chain. Let {*A*(*p*), *p*∈*ψ*} be the stochastic process of the discrete events corresponding to the Markov chain, *N* be the finite set of the solutions, and *ψ* be the set of indices for different stages of the stochastic process. The Markov chain highlights that, historical dynamic actions share the same features as future dynamic actions. In our problem, the identicalness of the features manifests as follows: the state of urban ecological efficiency A in year *p* + 1 directly bears on the probability for *A* to belong to type *j* in year *p*. Let *GR*_*ij*_ be the probability for urban ecological efficiency belonging to type *i* in year *p* to transfer to type *j* in year *p* + 1; that is, the maximum likelihood estimation of the transition is *GR*_*ij*_ = *m*_*ij*_/*m*_*i*_; let *m*_*ij*_ be the number of cities shifting from type *k* in year *p* to type *j* in year *p* + 1 and *m*_*i*_ be the total number of cities belonging to type *i* in the study period. Then, we have

(14) $$\begin{array}{c} GR\left\{A_{p}=j|A_{p-1}=i,A_{p-2}=i_{p-2},A_{0}=i_{0}\right\}=GR\left\{A_{m}=j|A_{m-1}=i\right\}=GR_{ij}. \end{array}$$

By dividing urban ecological efficiencies in the region into *x* states, it is possible to obtain an *x* × *x* state transition probability matrix of the Markov chain.

## 3. Multiobjective Optimal Decision-Making Model

### 3.1. Preliminaries

**Figure 1:**
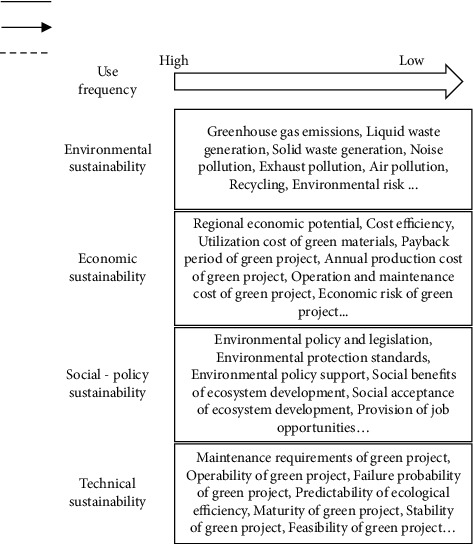
Evaluation attributes for the sustainable development of regional ecosystem.

Figure 1 summarizes the evaluation attributes for the sustainable development of the regional ecosystem. The evaluation system for the sustainable development of the regional ecosystem involves multiple dimensions, such as environmental sustainability, economic sustainability, social policy sustainability, and technical sustainability. The evaluation dimensions can all be quantified. For the sustainable development of the regional ecosystem, decision makers can also set up individualized evaluation systems based on objective needs and subjective judgement.

**Figure 2:**
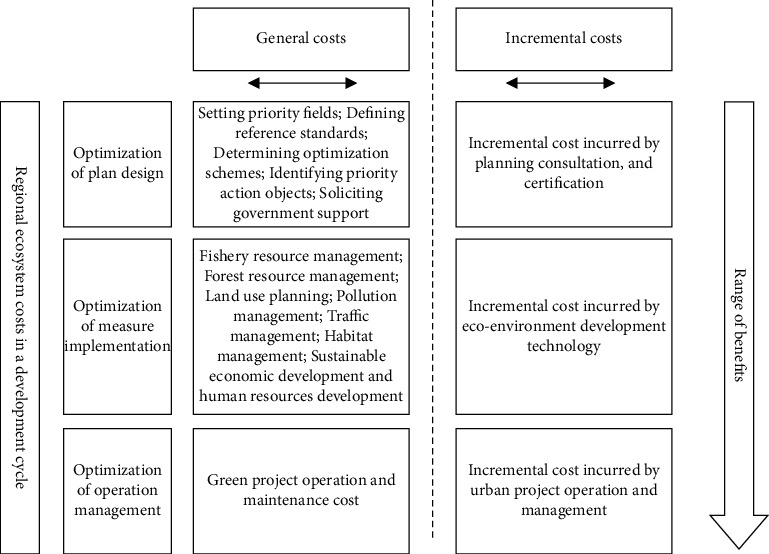
Costs and benefits of regional ecosystem in a development cycle.

To clarify the incremental costs and benefits of regional ecosystem development, this paper focuses on the incremental costs incurred during the optimization of plan design and measures implementation for regional ecosystem development. The costs of these two stages are mainly invested continuously by green project investors, developers, and implementers. The incremental benefits will continue to appear after the optimization measures are implemented in the regional ecosystem.  Figure 2 details the costs and benefits of the regional ecosystem in a development cycle.

Our problem is to sustainably improve the regional ecosystem, that is, combining and optimizing multiple optional measures for the ecosystem optimization to realize multiple goals under specific constraints, in reference to multiple sets of environmental big data in different periods, such that the optimized regional ecosystem is much more sustainable than the original system. This paper builds a weighted multiobjective optimization model to improve the sustainability of the regional ecosystem in multiple dimensions, relying on optimization measures. On this basis, the authors strived to make sustainable intelligent decisions.

### 3.2. Model Construction and Solving

**Figure 3:**
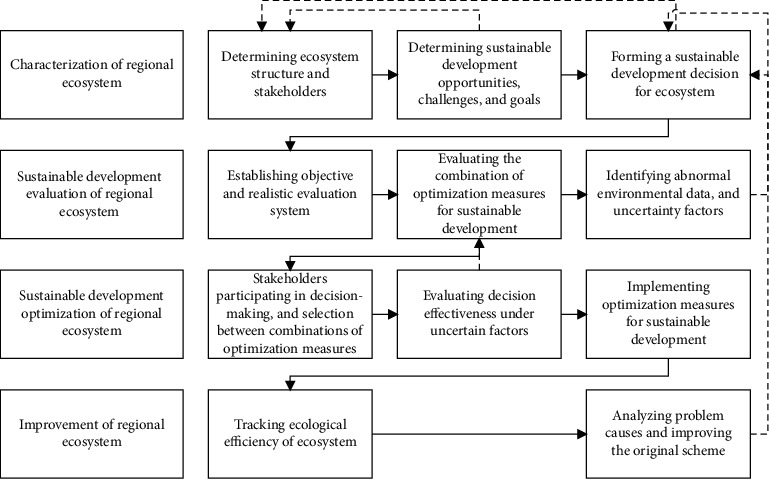
Improvement of sustainable development efficiency.

Targeting the regional ecosystem, our model tries to improve the ecological efficiency of the ecosystem by adopting the improved combination of optimization measures for sustainable development.  Figure 3 provides the specific steps to improve sustainable development efficiency: characterization of the regional ecosystem, sustainable development evaluation of REE, sustainable development optimization of REE, and sustainable development improvement of REE.

**Figure 4:**
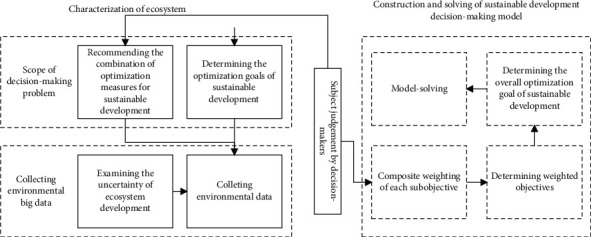
Multiobjective optimal decision-making model for the sustainable development of regional ecosystem.

The optimal decision making for the sustainable development of REE involves multiple steps and goals in four dimensions, including environmental sustainability, economic sustainability, social policy sustainability, and technical sustainability. The decision-making process is complicated by the diverse constraints on the different goals and the interference of uncertain decision-making factors. Hence, this paper presents a multiobjective optimal decision-making model (Figure 4) for the sustainable development of the regional ecosystem. The proposed model consists of two parts, namely, the characterization of the ecosystem, and construction and solving of sustainable development decision-making model and opens the path to improve the sustainable development of the regional ecosystem through spatiotemporal evolution.

The proposed multiobjective optimal decision-making model is targeted at regional ecosystems, whose ecological efficiencies obey different spatial distributions. The model was constructed through composite weighting of each subobjective, setting weighted objectives and sustainable objectives, and solving the model. Firstly, the interval analytic hierarchy process (IAHP) was adopted to assign composite weight to each objective, eliminating the need to consider the interaction between subobjectives. The composite weighting begins with the construction of an interval comparison matrix.

The subjective weight of each subobjective was quantified through expert judgement, which is uncertain to a certain extent. Therefore, the comparison matrix is composed of the following interval numbers:

(15) $$\begin{array}{c} F=\left[\begin{array}{cccc} \left[1,1\right] & \left[f_{12}^{K},f_{12}^{V}\right] & \cdots & \left[f_{1m}^{K},f_{1m}^{V}\right]\\ \left[f_{21}^{K},f_{21}^{V}\right] & \left[1,1\right] & \cdots & \left[f_{2m}^{K},f_{2m}^{V}\right]\\ \vdots & \vdots & \ddots & \vdots \\ \left[f_{m1}^{K},f_{m1}^{V}\right] & \left[f_{m2}^{K},f_{m2}^{V}\right] & \cdots & \left[1,1\right] \end{array}\right], \end{array}$$

where *m* is the number of optimization subobjectives; [*f*_*ij*_^*K*^, *f*_*ij*_^*V*^] is the relative importance of subobjective *i* to subobjective *j*, both of which are expressed as interval numbers. Next, the interval comparison matrix F was converted into the interval priority matrix *S*:

(16) $$\begin{array}{c} S=\left[\begin{array}{cccc} \left[0.5,0.5\right] & \left[s_{12}^{K},s_{12}^{V}\right] & \cdots & \left[s_{1m}^{K},s_{1m}^{V}\right]\\ \left[s_{21}^{K},s_{21}^{V}\right] & \left[0.5,0.5\right] & \cdots & \left[s_{2m}^{K},s_{2m}^{V}\right]\\ \vdots & \vdots & \ddots & \vdots \\ \left[s_{m1}^{K},s_{m1}^{V}\right] & \left[s_{m2}^{K},s_{m2}^{V}\right] & \cdots & \left[0.5,0.5\right] \end{array}\right], \end{array}$$

where

(17) $$\begin{array}{c} s_{ij}^{-}=\frac{f_{ij}^{-}}{f_{ij}^{-}+f_{ij}^{+}},s_{ij}^{+}=\frac{f_{ij}^{+}}{f_{ij}^{-}+f_{ij}^{+}}. \end{array}$$

After that, the subjective weights were calculated. Let *d*_*i*_^*V*^, *d*_*i*_^*K*^, *c*_*i*_^*V*^, and *c*_*i*_^*K*^ be the positive and negative biases in objective programming, respectively; [*υ*_*i*_^*K*^, *υ*_*i*_^*V*^] be the interval value range of the relative importance for each subobjective corresponding to the optimal solution. Then, the optimal solution of matrix *S* can be obtained by

(18) $$\begin{array}{c} \text{Min}\delta =\sum_{i=1}^{m}\left(d_{i}^{V}+d_{i}^{K}+c_{i}^{V}+c_{i}^{K}\right),\\ \text{s.t.}\upsilon_{i}^{K}+\sum_{j=1,j\neq i}^{m}\upsilon_{i}^{V}\geq 1,\\ \upsilon_{i}^{U}+\sum_{j=1,j\neq i}^{m}\upsilon_{i}^{K}\leq 1,\\ \;0\leq \upsilon_{i}^{K}\leq \upsilon_{i}^{V}\leq 1,\\ \left(\sum_{j=1}^{m}s_{ij}^{K}-m+0.5\right)\upsilon_{i}^{K}+\sum_{j=1,j\neq i}^{m}r_{ij}^{K}\upsilon_{i}^{V}-d_{i}^{V}+d_{i}^{K}=0,\\ \left(\sum_{j=1}^{m}s_{ij}^{V}-m+0.5\right)\upsilon_{i}^{V}+\sum_{j=1,j\neq i}^{m}s_{ij}^{V}\upsilon_{i}^{K}-c_{i}^{V}+c_{i}^{K}=0,\\ d_{i}^{V},d_{i}^{K},c_{i}^{V},c_{i}^{K}\geq 0. \end{array}$$

The subjective weight of each objective can be obtained by

(19) $$\begin{array}{c} q_{i}^{e}=\frac{\left(\upsilon_{i}^{K}+\upsilon_{i}^{V}\right)}{\sum_{i=1}^{m}\left(\upsilon_{i}^{K}+\upsilon_{i}^{V}\right)}. \end{array}$$

The overall objective for the sustainable development of the regional ecosystem combines multiple subobjectives, which are impossible to achieve all at once. To realize the overall objective, the traditional subobjective setting approach needs to adjust the subobjectives repeatedly. Based on multiple sets of environmental big data on the optimization system in different periods, this paper puts forward a novel weighted subobjective setting strategy.

According to the weights of subobjectives for the sustainable development of the regional ecosystem as well as the sustainable performance of each subobjective reflected by the multiple sets of environmental big data, the weighted subobjectives can be configured under a constraint as follows:

(20) $$\begin{array}{c} \max SG_{i}^{*}=q_{i}\times \left[\sum_{j\in T}\left(\psi_{j}\times \bar{\gamma_{ij}^{V}}\right)\right],\\ \text{s.t.}\psi_{j}\left(1-\psi_{j}\right)=0,T\in D, \end{array}$$

where *γ*_*ij*_^*V*^ be the performance value of subobjective *i* reflected by the normalized environmental sample data *j* (If *ψ*_*j*_ = 1 or 0 in *ψ*_*j*_(1 − *ψ*_*j*_) = 0, then the *j*-th set of environmental sample data can or cannot serve as a reference); *D* is the constraint; *T* ∈ *D* is the requirement that any combination of optimization measures must satisfy *D*.

Through the above steps, all weighted subobjectives can be obtained as *SG*^*∗*^=[*SG*_1_^*∗*^, *SG*_2_^*∗*^,…, *SG*_*m*_^*∗*^]. Each weighted subobjective corresponds to a specific model. Each combination *T*_*i*_^*∗*^ of optimization measures leads to the optimal solution to each specific model.

This paper likens the set of optimal solutions on all weighted subobjectives as the ideal solution to the objective of sustainable development, which can be vectorized as

(21) $$\begin{array}{c} \overset{⟶}{E}\left(JL\right)=\left[SG_{1}^{*},SG_{2}^{*},\ldots ,SG_{m}^{*}\right]. \end{array}$$

The ideal solution above can be transformed to an actual solution under the condition *T*_1_^*∗*^=*T*_2_^*∗*^=⋯=*T*_*m*_^*∗*^. For any feasible solution, the corresponding combination of optimization measures *T*_*b*_ can be vectorized as

(22) $$\begin{array}{c} \vec{E}\left(T_{b}\right)=\left[\overset{⟶}{E}\left(T_{b}^{K}\right),\overset{⟶}{E}\left(T_{b}^{V}\right)\right],\\ =\left\{\left[SG_{1}\left(T_{b}^{K}\right),SG_{1}\left(T_{b}^{V}\right)\right],\left[SG_{2}\left(T_{b}^{K}\right),SG_{2}\left(T_{b}^{V}\right)\right],\ldots ,\left[SG_{m}\left(T_{b}^{K}\right),SG_{m}\left(T_{b}^{V}\right)\right]\right\}. \end{array}$$

Considering the length and direction of vector functions, the degree of absolute improvement of sustainable development in the ideal solution IS can be given by

(23) $$\begin{array}{c} N\left(\text{IS}\right)=\left\|\overset{⟶}{E}\left(\text{IS}\right)\right\|=\sqrt{\sum_{i=1}^{m}{\left(SG_{i}^{*}\right)}^{2}}. \end{array}$$

The degree of absolute improvement of sustainable development in the feasible solution *b* can be given by

(24) $$\begin{array}{c} N\left(T_{b}\right)=\left[N\left(T_{b}^{K}\right),N\left(T_{b}^{V}\right)\right]=\left[\left\|\overset{⟶}{E}\left(T_{b}^{K}\right)\right\|,\left\|\overset{⟶}{E}\left(T_{b}^{V}\right)\right\|\right],\\ =\left[\sqrt{\sum_{i=1}^{m}{\left(SG_{i}\left(T_{b}^{K}\right)\right)}^{2}},\sqrt{\sum_{i=1}^{m}{\left(SG_{i}\left(T_{b}^{V}\right)\right)}^{2}}\right]. \end{array}$$

The equilibrium of relative development of multiple weighted subobjectives in the ideal solution IS can be given by

(25) $$\begin{array}{c} \cos\left(F\left(\text{IS}\right)\right)=\left(\frac{\overset{⟶}{E}\left(\text{IS}\right)\cdot \overset{⟶}{E}\left(\text{IS}\right)}{\left\|\overset{⟶}{E}\left(\text{IS}\right)\right\|\cdot \left\|\overset{⟶}{E}\left(\text{IS}\right)\right\|}\right)=1. \end{array}$$

The equilibrium of relative development of multiple weighted subobjectives in the feasible solution *b* can be given by

(26) $$\begin{array}{c} \cos\left(F\left(T_{b}\right)\right)=\left[\cos\left(F\left(T_{b}^{K}\right)\right),\;\cos\left(F\left(T_{b}^{V}\right)\right)\right]=\left[\min\left\{\left(\frac{\overset{⟶}{E}\left(\text{IS}\right)\cdot \overset{⟶}{E}\left(T_{b}^{K}\right)}{\left\|\overset{⟶}{E}\left(\text{IS}\right)\right\|\cdot \left\|\overset{⟶}{E}\left(T_{b}^{K}\right)\right\|}\right),\left(\frac{\overset{⟶}{E}\left(\text{IS}\right)\cdot \overset{⟶}{E}\left(T_{b}^{V}\right)}{\left\|\overset{⟶}{E}\left(\text{IS}\right)\right\|\cdot \left\|\overset{⟶}{E}\left(T_{b}^{V}\right)\right\|}\right)\right\}\right.,\\ \max\left.\left\{\left(\frac{\overset{⟶}{E}\left(IS\right)\cdot \overset{⟶}{E}\left(T_{b}^{K}\right)}{\left\|\overset{⟶}{E}\left(IS\right)\right\|\cdot \left\|\overset{⟶}{E}\left(T_{b}^{K}\right)\right\|}\right),\left(\frac{\overset{⟶}{E}\left(IS\right)\cdot \overset{⟶}{E}\left(T_{b}^{V}\right)}{\left\|\overset{⟶}{E}\left(IS\right)\right\|\cdot \left\|\overset{⟶}{E}\left(T_{b}^{V}\right)\right\|}\right)\right\}\right]=\left[\min\left\{\left(\frac{\sum_{i=1}^{m}\left[SG_{i}^{*}\times SG_{i}\left(T_{b}^{K}\right)\right]}{\sqrt{\sum_{i=1}^{m}{\left(SG_{i}^{*}\right)}^{2}}\times \sqrt{\sum_{i=1}^{m}{\left[SG_{i}\left(T_{b}^{K}\right)\right]}^{2}}}\right),\left(\frac{\sum_{i=1}^{m}\left[SG_{i}^{*}\times SG_{i}\left(T_{b}^{V}\right)\right]}{\sqrt{\sum_{i=1}^{m}{\left(SG_{i}^{*}\right)}^{2}}\times \sqrt{\sum_{i=1}^{m}{\left[SG_{i}\left(T_{b}^{V}\right)\right]}^{2}}}\right)\right\},\max\left\{\left(\frac{\sum_{i=1}^{m}\left[SG_{i}^{*}\times SG_{i}\left(T_{b}^{K}\right)\right]}{\sqrt{\sum_{i=1}^{m}{\left(SG_{i}^{*}\right)}^{2}}\times \sqrt{\sum_{i=1}^{m}{\left[SG_{i}\left(T_{b}^{K}\right)\right]}^{2}}}\right),\left(\frac{\sum_{i=1}^{m}\left[SG_{i}^{*}\times SG_{i}\left(T_{b}^{V}\right)\right]}{\sqrt{\sum_{i=1}^{m}{\left(SG_{i}^{*}\right)}^{2}}\times \sqrt{\sum_{i=1}^{m}{\left[SG_{i}\left(T_{b}^{V}\right)\right]}^{2}}}\right)\right\}\left(\right]\right]. \end{array}$$

By the principle of vector projection, the equilibrium of relative development of weighted subobjectives can be unified with the absolute improvement of sustainable development. In ideal conditions, the optimal degree of improvement of regional sustainable development can be given by

(27) $$\begin{array}{c} SP\left(\text{IS}\right)=\left[N\left(\text{IS}\right)\times \cos\left(F\left(\text{IS}\right)\right)\right]=\sqrt{\sum_{i=1}^{m}{\left(SG_{i}^{*}\right)}^{2}}. \end{array}$$

The improvement effect of the feasible solution *T*_*b*_ on sustainable development can be quantified by

(28) $$\begin{array}{c} SP\left(T_{b}\right)=\left[SP\left(T_{b}^{K}\right),SP\left(T_{b}^{V}\right)\right]=\left[N\left(T_{b}^{K}\right)\times \cos\left(F\left(T_{b}^{K}\right)\right),N\left(T_{b}^{V}\right)\times \cos\left(F\left(T_{b}^{V}\right)\right)\right]. \end{array}$$

The intelligent decision making of the objective of sustainable development can be derived from formulas (27) and (28). To display the decision-making process more intuitively, an objective achievement rate was introduced to transform the two formulas:

(29) $$\begin{array}{c} \text{OBJ}\left(T_{l}\right)=\left[\text{OBJ}\left(T_{l}^{K}\right),\text{OBJ}\left(T_{l}^{V}\right)\right]=\left[\frac{SP\left(T_{l}^{K}\right)}{SP\left(IS\right)},\frac{SP\left(T_{l}^{V}\right)}{SP\left(IS\right)}\right]\times 100\%. \end{array}$$

The above formula shows that the objective achievement rate of IS is 1 and that of *T*_*b*_ falls in between (0, 1) (the closer the value is to 1, the better the optimization effect). Through the above analysis, the overall optimization objective of sustainable development for the regional ecosystem can be expressed as

(30) $$\begin{array}{c} \text{Max}\text{OBJ}\left(T_{l}\right),\\ \text{s.t.}T_{l}\in D. \end{array}$$

The above formula also outputs an interval number. Under uncertainty conditions, the model can be solved by probability comparison:

(31) $$\begin{array}{c} S_{\left(a>t\right)}=max\left\{1-max\left(\frac{W_{t}^{V}-W_{a}^{K}}{W_{t}^{V}-W_{a}^{K}+W_{t}^{V}-W_{a}^{K}},0\right),0\right\}. \end{array}$$

Any interval numbers *W*_*t*_=[*W*_*t*_^*K*^, *W*_*t*_^*V*^] and *W*_*a*_=[*W*_*a*_^*K*^, *W*_*a*_^*V*^] can be compared in terms of probability. If *W*_*a*_ > *W*_*t*_, i.e., *S*_(*a*>*t*)_, *S*_(*a*>*t*)_ > 0.5 means *W*_*a*_ is probably better than *W*_*t*_; the inverse is also true. Similarly, if *W*_*t*_^*V*^ < *W*_*a*_^*V*^ and *W*_*t*_^*K*^ < *W*_*a*_^*K*^, *W*_*a*_ must be better than *W*_*t*_.

## 4. Experiments and Result Analysis

**Figure 5:**
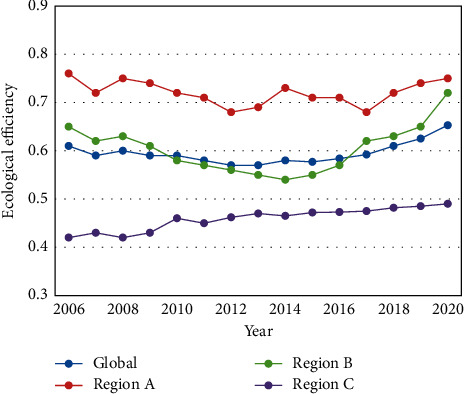
REE trends in 2005–2020.

Figure 5 presents the evolution trends of the overall ecological efficiency of the study area in 2005–2020. The REEs exhibited obvious features of phased development, dropping from 0.6024 in 2006 to the lowest point of 0.5785 in 2013. From 2014 to 2020, the REEs rose significantly and peaked at 0.6549. The reasons for the phased development are as follows:

In the early phase of the sample period, the regional economy developed extensively in the traditional model, and the society had poor awareness of ecoenvironmental protection. That is why the REEs in the study area continued to decline. In 2013–2014, the cities in the study area quickly adjusted their development pattern and publicized ecological civilization, which contribute to the continuous growth of REEs.

There were some differences between regions in ecological efficiency. Region A, a transportation hub in a strategic location, remained the leader of ecological efficiency throughout the 15-year-long sample period. The ecological efficiencies of Regions A and B oscillated similarly as the overall ecological trend of the study area. After 2015, the ecological efficiency of Region B increased markedly each year, with increasingly strong momentum. Region C had the lowest ecological efficiency, which grew stably over the 15 years.

**Figure 6:**
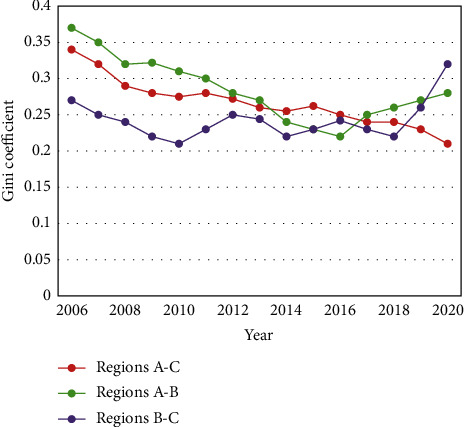
Trends of regional REE differences.

Figure 6 compares the REE evolution trends of each pair of the three regions. Through the sample period, the largest regional difference in ecological efficiency existed between Regions A and B, whose mean Gini coefficient was 0.274. The second-largest regional difference was observed between Regions A and C, whose mean Gini coefficient was 0.261. The smallest regional difference was observed between Regions B and C, whose mean Gini coefficient was 0.253.

In general, the ecological efficiency gap between Regions A and C continued to narrow at an annual change rate of −2.14%. The gap between Regions B and C widened after 2010, despite some fluctuations, with an annual change rate of 0.87%. Specifically, the gap between Regions A and C declined with fluctuations from 0.347 in 2006 to 0.206 in 2020, reaching the valley of the sample period. The gaps between Regions A and B, and between Regions B and C both decreased first and then increased. The gap between Regions A and B minimized at 0.223 in 2016, while that between Regions B and C minimized at 0.204 in 2010.

To sum up, the relevant government departments should strengthen the coordination between Regions B and C in green development and reduce the spatial difference between regions in ecological efficiency without ignoring the regional difference between A and B.

Through Gini coefficient decomposition, this paper looks for the sources of REE spatial differences, that is, analyzes the contributions of intraregion difference, inter-regional difference, and transvariation intensity to overall regional spatial differences.  Table 2 presents the calculated results on intra and inter-regional contributions.

Through the sample period, the inter-regional difference of A and B contributed the greatest to the spatial gap of REE, followed in turn by that of B and C, and that of A and C; the intraregional difference of C contributed the greatest to the spatial gap of REE, followed in turn by that of A and that of B. It can be observed that the spatial imbalance of REEs can be effectively solved by reducing inter-regional differences. Regions A and B should be the focus of regulation because their inter-regional difference is the major contributor to the spatial gap of REE.

Tables 3–5 report the transition probability matrices of the Markov chain for Regions A–C, respectively. Note that period *p*_1_ has a one-year lag, and period *p*_2_ has a two-year lag. The long-term transition trend of REE can be derived from these matrices. In each matrix, the elements to the left of the diagonal were smaller than those to the right, indicating that the overall ecological efficiency of each region moves clearly from the low level to the high level. Meanwhile, the REE had a greater stable probability than transition probability. This means that the state transition of ecological efficiency is accompanied by apparent polarization. As period *p*_1_ changed to period *p*_2_, i.e., the lag increased from 1 year to 2 years, the overall ecological efficiency of the study area basically remained the same, with a very slight decline. In addition, there were many nonzero elements on both sides of the diagonal in each matrix, suggesting the difficulty for the study area to realize the leapfrog transition of ecological efficiency.

**Figure 7:**
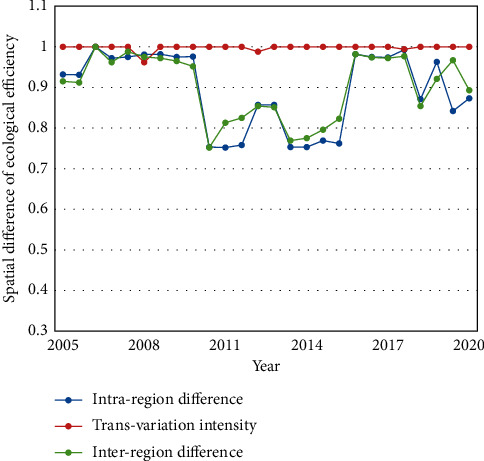
REE decomposition and changes in 2005–2020.

This paper employs the multistage method to measure the contributions of intraregion difference, inter-regional difference, and transvariation intensity in 2005–2020. The results are presented in Table 6 and Figure 7. The measured results of our method have a smaller variation than those of the traditional DEA. The following can be inferred from the data in Table 6. In 2005–2020, the mean contribution was 0.813, 0.990, and 0.821 for intraregional difference, inter-regional difference, and transvariation intensity, respectively. Overall, the study area had relatively high ecological efficiency since 2005. The regional economic development achieves a high cost effectiveness in terms of energy-saving technology and green environmental investment. In 2010 and 2013, the contribution of inter-regional difference was merely 0.758 and 0.761, respectively. In 2009–2011 and 2013–2016, the inter-region contributions were below the mean of the 15 years. Through the 15-year-long sample period, the transvariation intensity made relatively high contributions, reflecting the good effect of technical and management measures adopted by the regional ecosystem.

## 5. Conclusions

Based on the analysis of environmental big data, this paper investigates the evaluation of REE and intelligent decision support for sustainable development. Specifically, the spatiotemporal evolution of REE was analyzed based on environmental big data, and the spatial layout of REE and the sources of the spatial differences were both clarified. In addition, the authors constructed a multiobjective optimal decision-making model for the sustainable development of a regional ecosystem and presented the solving method for the model. After that, experiments were carried out to analyze the REE changes and inter-regional REE differences in 2005–2020. The experimental results demonstrated the feasibility of our analytical method. Furthermore, the authors discussed the contributions of intraregional difference, inter-regional difference, and transvariation intensity to the overall spatial difference of regional ecological efficiency, constructed the transition probability matrix of the Markov chain for the REE in each region, and provided the measured results on regional REEs.

The future research will try to develop a decision model capable of considering and solving different interest preferences and demands of different decision makers and realize multirole, multiattribute decision making as well as multirole, multiobjective decision making.

## Figures and Tables

**Table 1:** REE evaluation based on environmental big data.

Environmental type	Parameters	Influencing factors of ecological efficiency
Building area	Newly built area, energy consumption per unit area, and energy consumption structure	Energy-efficient design, building energy conservation and emission reduction, and green building material utilization
Traffic area	Passenger capacity per unit time	Public transit travel rate and green travel rate
Water area	Water supply and sewage treatment capacity	Water-saving equipment usage, water-saving indices, and wastewater and sewage reuse rate
Landscape area	Green area and greening rate	Vegetation coverage and regional carbon sequestration capacity

**Table 2:** Source of differences and contributions.

Year	Intraregional contribution			Inter-regional contribution		
	Region A	Region B	Region C	Region A	Region B	Region C
2005	12.36	11.95	6.35	7.86	17.38	11.24
2006	13.52	11.36	5.41	10.25	16.23	12.08
2007	14.75	10.27	5.23	11.75	19.75	10.61
2008	15.23	8.62	5.75	13.28	21.61	10.25
2009	14.76	10.15	5.76	10.94	20.85	14.31
2010	14.76	10.36	5.23	8.51	17.23	14.52
2011	15.23	10.62	4.81	8.72	16.05	11.23
2012	15.08	13.54	5.08	7.63	17.28	13.54
2013	13.27	15.76	3.64	3.55	15.36	17.21
2014	10.75	13.28	4.76	3.42	17.54	20.36
2015	11.25	11.39	4.52	0.46	18.95	20.85
2016	13.62	14.25	4.08	5.23	21.72	28.30
2017	11.22	13.21	2.59	7.41	19.35	27.26
2018	9.35	15.02	2.79	2.88	20.08	25.42
2019	11.76	11.53	1.26	6.58	22.72	27.36
2020	10.05	9.74	2.65	6.75	23.45	31.65
Mean	12.94	11.94	4.37	7.21	19.09	14.76

**Table 3:** Transition probability matrix of the Markov chain for Region A.

Period	*p*_1_				*p*_2_			
Type	1	2	3	4	1	2	3	4
1	0.7235	0.1122	0	0	0.6758	0.1523	0	0
2	0.2753	0.6235	0.0851	0	0.1532	0.5123	0.1231	0
3	0	0.2753	0.6723	0.1123	0	0.3154	0.5876	0.1531
4	0	0	0.2235	0.8517	0	0	0.2753	0.8675

**Table 4:** Transition probability matrix of the Markov chain for region B.

Period	*p*_1_				*p*_2_			
Type	1	2	3	4	1	2	3	4
1	0.5321	0.0223	0	0	0.3725	0.0275	0	0
2	0.4233	0.4555	0.0853	0.0153	0.5742	0.0375	0.0723	0.0181
3	0.0257	0.4125	0.0545	0.1535	0.0436	0.3122	0.4368	0.2376
4	0.0257	0.1333	0.3721	0.8755	0.0214	0.2763	0.4214	0.7532

**Table 5:** Transition probability matrix of the Markov chain for region C.

Period	*p*_1_				*p*_2_			
Type	1	2	3	4	1	2	3	4
1	0.6355	0.1321	0	0	0.5728	0.0972	0	0
2	0.3721	0.5765	0.1123	0	0.3721	0.5321	0.1423	0.0354
3	0.0253	0.2675	0.5721	0.2222	0.0434	0.3122	0.5472	0.2235
4	0	0.0223	0.3555	0.7655	0	0.0251	0.3222	0.7652

**Table 6:** Overall ecological efficiencies of the study area in 2005–2020.

Year	Intraregional difference	Inter-region difference	Transvariation intensity
2005	0.932	0.915	1
2006	1	1	1
2007	0.975	0.962	1
2008	0.982	0.988	0.962
2009	0.976	0.975	1
2010	0.752	0.752	1
2011	0.857	0.813	1
2012	0.753	0.756	1
2013	0.769	0.769	0.988
2014	0.982	0.982	1
2015	0.974	0.974	1
2016	0.993	0.993	1
2017	0.871	0.854	1
2018	0.963	0.921	0.994
2019	0.842	0.867	1
2020	0.873	0.893	1

## Data Availability

The data used to support the findings of this study are available from the corresponding author upon request.
